# False Positive Findings on I-131 WBS and SPECT/CT in Patients with History of Thyroid Cancer: Case Series

**DOI:** 10.1155/2017/8568347

**Published:** 2017-01-26

**Authors:** Zeina C. Hannoush, Juan D. Palacios, Russ A. Kuker, Sabina Casula

**Affiliations:** ^1^Division of Endocrinology, Diabetes and Metabolism, Department of Medicine, University of Miami Miller School of Medicine, Miami, FL 33136, USA; ^2^Division of Nuclear Medicine, Department of Radiology, University of Miami Miller School of Medicine, Miami, FL 33136, USA

## Abstract

*Introduction.* Although whole body scan (WBS) with I-131 is a highly sensitive tool for detecting normal thyroid tissue and metastasis of differentiated thyroid cancer (DTC), it is not specific. Additional information, provided by single photon emission computed tomography combined with X-ray computed tomography (SPECT/CT) and by the serum thyroglobulin level, is extremely useful for the interpretation of findings.* Case Presentation.* We report four cases of false positive WBS in patients with DTC: ovarian uptake corresponding to an endometrioma, scrotal uptake due to a spermatocele, rib-cage uptake due to an old fracture, and hepatic and renal uptake secondary to a granuloma and simple cyst, respectively.* Conclusions.* Trapping, organification, and storage of iodine are more prominent in thyroid tissue but not specific. Physiologic sodium-iodine symporter expression in other tissues explains some, but not all, of the WBS false positive cases. Other proposed etiologies are accumulation of radioiodine in inflamed organs, metabolism of radiodinated thyroid hormone, presence of radioiodine in body fluids, and contamination. In our cases nonthyroidal pathologies were suspected since the imaging findings were not corroborated by an elevated thyroglobulin level, which is considered a reliable tumor marker for most well-differentiated thyroid cancers. Clinicians should be aware of the potential pitfalls of WBS in DTC to avoid incorrect management.

## 1. Introduction

In thyroidectomized patients with history of well-differentiated thyroid cancer (DTC), scintillation scanning of the whole body (WBS) after either a diagnostic or therapeutic dose of radioactive iodine administration is considered the routine method to identify the physical location of local or distant, iodine avid, metastasis [1]. This relies on the fact that most of the DTC cells retain the property, unique to the thyroid epithelial follicular cells, to concentrate, organify, and accumulate iodine through the action of the sodium-iodine symporter (NIS) [2]. Although WBS is highly sensitive for detecting thyroid tissue, it is not very specific; therefore, false positive images can be seen in clinical practice and their recognition is critical for correct management [3, 4].

Single photon emission computed tomography (SPECT) combined with X-ray computed tomography (CT) is a sophisticated adjuvant tool that has shown to be useful for better localization of lesions seen on planar views [5]. Here we describe four clinical cases with positive distant uptake, where SPECT/CT played an essential role in the localization and interpretation of the results seen on WBS. Nonthyroidal pathologies were suspected since the imaging findings were not corroborated by an elevated level of serum thyroglobulin, which in the absence of thyroglobulin antibodies is considered a sensitive and reliable tumor marker for most well-differentiated thyroid cancers. In these clinical scenarios it is essential to avoid exposing the patients to unnecessary additional radioiodine treatment for presumptive metastasis.

## 2. Case Presentation

**Figure 1:**
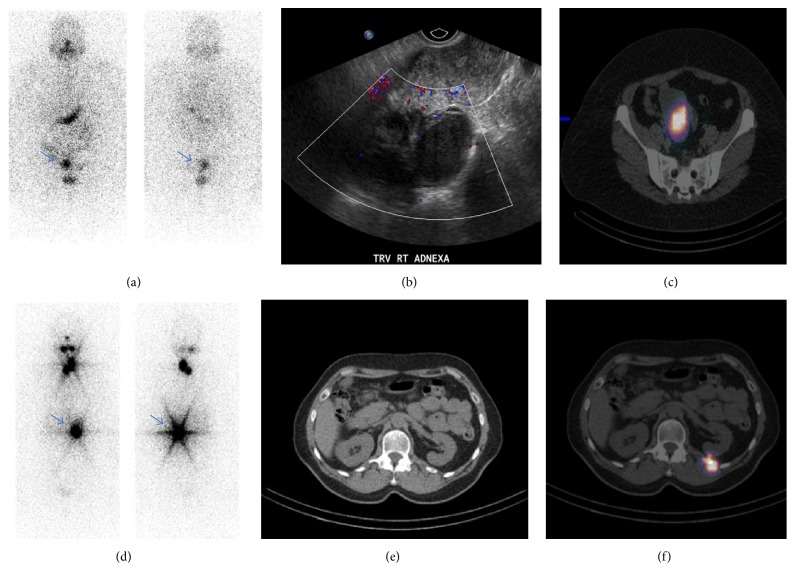
(a) Anterior and posterior planar views from a diagnostic I-131 WBS show focal uptake in the right pelvis (arrow). (b) Transverse sonographic image of the right adnexa shows a lobulated complex solid and cystic lesion with internal vascularity. (c) SPECT/CT localizes the area of radioiodine uptake to the right adnexal lesion, which on final pathology after surgical resection showed an endometrioma. (d) Anterior and posterior planar views from a postablation I-131 WBS show expected radiotracer uptake in the neck as well as an incidental focus of intense activity in the left lower thorax (arrow). (e) Axial CT image through the lower thorax shows a nondisplaced fracture of the left twelfth rib. (f) SPECT/CT localizes the area of radioiodine uptake to the left twelfth rib, which was biopsied and showed no evidence of malignancy.

*Case #1.* We present the case of a 46-year-old woman with history of stage T1N0M0 papillary thyroid cancer, mixed follicular and solid variant status after thyroidectomy followed by radioactive iodine (RAI) ablation therapy with 99 mCi of I-131. Initial WBS showed physiologic uptake in the thyroid bed. On the one-year post-treatment surveillance workup, she was found to be in biochemical remission with a negative stimulated thyroglobulin level and negative thyroglobulin antibodies. However, the I-131 WBS demonstrated an area of iodine accumulation in the pelvis above the urinary bladder, which localized to the right adnexa on SPECT/CT. Pelvic ultrasound revealed an 8 × 8 × 5 cm hypoechoic vascular and lobulated mass in the right adnexal region. For that reason, the patient underwent hysterectomy with right oophorectomy. The pathology was consistent with endometrioma [Figures 1(a)–1(c)].

*Case #2*. We present the case of a 63-year-old woman with history of stage T1bN1Mx papillary thyroid cancer, follicular and classical variant status after thyroidectomy followed by RAI ablation therapy with 28 mCi of I-131. The post-treatment WBS revealed uptake in the neck, and a separate focus of intense uptake in the left lower thorax, which localized to the left 12th rib on SPECT/CT. A subsequent bone scan confirmed the presence of an osteoblastic lesion in the left 12th rib suspicious for metastasis. The stimulated thyroglobulin at the time she received her ablation treatment was 7.2 ng/mL with negative thyroglobulin antibodies, more consistent with thyroid remnant than distant metastasis. She underwent biopsy of the suspicious bone lesion and pathology was negative for malignancy. CT scan images revealed a rib fracture at that level (Figures 1(d)–1(f)). 

**Figure 2:**
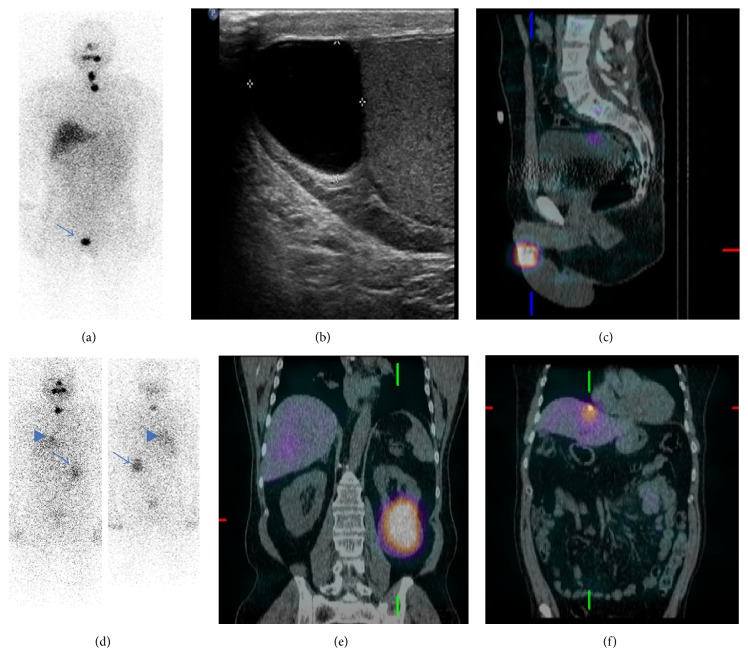
(a) Anterior planar view from a postablation I-131 WBS shows satisfactory targeting of radiotracer in the neck with an additional incidental focus of activity in the region of the right scrotum. (b) Sagittal sonographic image of the right scrotum shows a hypoechoic avascular lesion in the right epididymal head likely representing a spermatocele. (c) SPECT/CT localizes the area of radioiodine uptake to the right epididymal lesion. (d) Anterior and posterior planar views from a postablation I-131 WBS show expected radiotracer uptake in the neck as well as two incidental foci projecting over the dome of the liver (arrowhead) and in the left lower quadrant of the abdomen (arrow). (e) SPECT/CT localizes the area of radioiodine uptake to an exophytic cyst arising from the inferior pole of the left kidney. (f) SPECT/CT localizes the second radioiodine avid focus to a calcified granuloma in the dome of the liver.

*Case #3*. We present the case of a 49-year-old man with history of papillary thyroid cancer, classical variant status after thyroidectomy and RAI ablation therapy with 138 mCi of I-131. His thyroglobulin level was unreliable due to the presence of thyroglobulin antibodies. The posttherapy WBS showed uptake in the thyroid bed and in the cervical region compatible with a lymph node. There was an additional focus of iodine uptake in the right lower pelvis that localized above the right testicle on SPECT/CT. A scrotal ultrasound found no evidence of testicular abnormalities, but there was an interval increase in size of a right side spermatocele, which corresponded to the abnormal uptake noted on the nuclear medicine examination (Figures 2(a)–2(c)). 

*Case #4*. We present the case of a 62-year-old man with history of stage T3N1Mx papillary thyroid cancer, classical variant status after thyroidectomy and RAI ablation therapy with 150 mCi of I-131. His stimulated thyroglobulin level was 7.3 ng/mL with negative thyroglobulin antibodies. Post-treatment WBS with SPECT/CT showed physiologic uptake in the area of the thyroid bed as well as focal accumulation of radioiodine in the abdomen likely corresponding to a granuloma in the liver and a simple cyst in the left kidney as demonstrated by a follow-up sonogram. The patient had negative viral hepatitis serology and normal liver function tests (Figures 2(d)–2(f)).

## 3. Discussion and Conclusions

Iodine (I), the oxidized form of I^−^, is an essential constituent of thyroid hormones which are phenolic rings joined by an ether link iodinated at 3 positions (3,5,3′-tri-iodo-L-thyronine, or T_3_) or 4 positions (3,5,3′,5′-tetra-iodo-L-thyronine or T_4_) [6]. The uptake of iodine through the basolateral membrane of the follicular thyroid cells is a key point in the biosynthesis of thyroid hormone. This process is mediated by the sodium-iodine symporter (NIS), a 13-transmembrane domain glycoprotein that relies on the sodium (Na^+^) electrochemical gradient created by the Na^+^/K^+^ ATPase and allows active concentration of iodine by electrogenic symport of sodium (2 : 1 Na^+^ to I^−^ stoichiometry). Trapping, organification, and storage of iodine are usually more prominent in functioning thyroid tissue than other organs. Based on that principle, radioactive iodine has been used for both diagnostic and therapeutic purposes in patients with DTC [7].

NIS is also expressed in several other differentiated epithelia where it is not regulated by thyroid stimulating hormone (TSH) like salivary glands, thymus, lacrimal glands, gastric mucosa, choroid plexus, and lactating mammary glands [6]. Functional NIS expression in normal tissues can explain some but not all of the cases of false positive results on WBS. Other etiologies that have been proposed are accumulation of radioiodine in inflamed organs, metabolism of radiodinated thyroid hormone, the presence of radioiodine in body fluids, and therefore contamination by physiologic secretions. Common sites of physiologic ectopic radioactive iodine uptake seen in clinical practice include parotiditis, maxillary abscess, thyroglossal ducts, bronchiectasis, hiatal hernia, esophageal retention, and sebaceous cysts [1, 7, 8].

A conventional planar I-131 WBS can be a very sensitive diagnostic tool; however, a lack of anatomical landmarks and the nonspecific uptake of radiotracer somewhat complicate the interpretation of the images [5]. Hybrid systems integrating SPECT with a conventional CT allow a fairly exact overlay of molecular and morphological information and are more accurate for diagnosis and staging than WBS alone [9, 10]. The SPECT/CT images obtained in our case series were performed on Symbia T16 scanner (Siemens Medical Solutions) using a 128 × 128 pixel matrix and 30 seconds per projection view. Standard reconstruction technique entailed iterative reconstruction with a CT-based attenuation correction algorithm applied to the SPECT images. The CT component is a 16-slice scanner with a 5 mm slice thickness and a pitch of 0.8.

Endometrioma as a cause of false positive WBS findings was first described in 2000 and only a few reported cases exist in the literature [11, 12]. Inflammation has been recognized as a common cause of false positive findings on WBS [1] and our second case is a salient demonstration of how this can be encountered in clinical practice. To the best of our knowledge, radioactive iodine accumulation in a spermatocele causing false positive findings in the pelvis on WBS has not been previously described in the literature.

Diffuse hepatic uptake after I-131 therapy has been previously reported and it is thought to be caused by hepatic metabolism of radioiodinated thyroid hormones released by remnant thyroid tissue. This pattern of activity should be distinguished from focal hepatic uptake [2]. Focal false positive iodine accumulation in the liver has been encountered in patients with biliary duct dilation [13]; this is thought to be due to biliary excretion of radiodinated metabolites. Only a few cases of false positive radioactive iodine accumulation in simple liver cysts, not associated with biliary duct dilation, have been reported and the pathophysiology behind this accumulation is not well understood [14–16]. Many cases of false positive uptake seen in renal cysts have been reported. There are various hypotheses of the pathophysiology behind uptake in renal cysts including an active secretory process by the renal tubules and communication between the cyst and the renal collecting system [17].

Interpreting correctly the WBS can be challenging and often times the final diagnosis requires additional tests including alternative imaging studies and occasionally tissue biopsy. The level of thyroglobulin and the exact location of the uptake determined by the SPECT/CT are helpful tools in the initial assessment of these patients. Based on that knowledge nonthyroidal pathologies were suspected in our patients. It is important to highlight that even though DTC is usually an indolent disease with a good prognosis, distant metastasis is always a potential complication that accounts for most of its disease specific mortality. The most common metastatic sites are in the lungs and bones but brain, breast, liver, kidney, muscle, skin, and other sites of metastasis have been described. For this reason these patients should continue to receive close follow-up and surveillance according to American Thyroid Association guidelines [18].

In three cases, the imaging findings were not corroborated by a reliable elevated level of serum thyroglobulin, which in the absence of thyroglobulin antibodies usually reflects the tumor burden in most cases of DTC [19]. The patient with accumulation of RAI above the right testicle had positive thyroglobulin antibodies known to interfere with the immunometric assay that was used, limiting the interpretation of the test. However, while a case of testicular metastasis from medullary thyroid cancer was previously described [20], to our knowledge, there are no reports of testicular metastasis from DTC. A scrotal ultrasound confirmed that the false positive uptake was due to a spermatocele.

The four cases reported illustrate how the accurate evaluation of radioiodine WBS is critical in the management of patients with thyroid cancer and how SPECT/CT can improve the localization and characterization of lesions. Clinicians involved in the management of DTC should be aware of the potential pitfalls of radioiodine scans and of the possible mechanisms involved to avoid incorrect management.
